# *In vitro* Antibacterial Activity of an FDA-Approved H^+^-ATPase Inhibitor, Bedaquiline, Against *Streptococcus mutans* in Acidic Milieus

**DOI:** 10.3389/fmicb.2021.647611

**Published:** 2021-02-25

**Authors:** Meng Zhang, Wenqian Yu, Shujing Zhou, Bing Zhang, Edward Chin Man Lo, Xin Xu, Dongjiao Zhang

**Affiliations:** ^1^Shandong Provincial Key Laboratory of Oral Tissue Regeneration, Shandong Engineering Laboratory for Dental Materials and Oral Tissue Regeneration, School and Hospital of Stomatology, Cheeloo College of Medicine, Shandong University, Jinan, China; ^2^Faculty of Dentistry, University of Hong Kong, Sai Ying Pun, Hong Kong; ^3^Department of Stomatology, Maternal and Child Health Hospital of Liaocheng City, Liaocheng, China

**Keywords:** caries, antibacteria activity, *Streptococcus mutans*, acid resistance bacteria, acidic environment

## Abstract

**Background:**

Dental caries is an acid-related disease. Current anti-caries agents mainly focus on the bacteriostatic effect in a neutral environment and do not target acid-resistant microorganisms related to caries in acidic milieus.

**Objectives:**

To assess the *in vitro* antibacterial activities of bedaquiline against oral pathogens in acidic milieus.

**Methods:**

*Streptococcus mutans*, *Streptococcus sanguinis*, and *Streptococcus salivarius* were used to prepare the mono-/multiple suspension and biofilm. The MIC and IC_50_ of bedaquiline against *S. mutans* were determined by the broth microdilution method. Bedaquiline was compared regarding (i) the inhibitory activity in pH 4–7 and at different time points against planktonic and biofilm; (ii) the effect on the production of lactic acid, extracellular polysaccharide, and pH of *S. mutans* biofilm; (iii) the cytotoxicity effects; and (iv) the activity on H^+^-ATPase enzyme of *S. mutans*.

**Results:**

In pH 5 BHI, 2.5 mg/L (IC_50_) and 4 mg/L (MIC) of bedaquiline inhibited the proliferation and biofilm generation of *S. mutans* and Mix in a dose-dependent and time-dependent manner, but it was invalid in a neutral environment. The lactic acid production, polysaccharide production, and pH drop range reduced with the incorporation of bedaquiline in a pH 5 environment. Its inhibitory effect (>56 mg/L) against H^+^-ATPase enzyme in *S. mutans* and its non-toxic effect (<10 mg/L) on periodontal ligament stem cells were also confirmed.

**Conclusion:**

Bedaquiline is efficient in inhibiting the proliferation and biofilm generation of *S. mutans* and other oral pathogens in an acidic environment. Its high targeting property and non-cytotoxicity also promote its clinical application potential in preventing caries. Further investigation of its specific action sites and drug modification are warranted.

## Introduction

Dental caries is one of the most common oral diseases worldwide that affect oral health and general health (Smith AGC Kassebaum et al., 2017; Peres et al., 2019). The capability of caries-related pathogens to continuously metabolize carbohydrate in the acidic environment that they gradually create has been considered to be closely related to the initial development of dental decay (Selwitz et al., 2007). Numerous antibacterial agents, including quaternary ammonium monomer (Zhang et al., 2015; Ibrahim et al., 2020), fluoridated silver (Yin et al., 2020), and nanoparticle materials (Gao et al., 2016; Benoit et al., 2019), have been explored trying to inhibit those caries-related bacteria and balance plaque microecology (Pereira-Cenci et al., 2013). However, the conundrum is that it is difficult to target only caries-related microorganisms without interfering with normal microflora.

Acid resistance is one of the vital cariogenic properties of caries-related microorganisms (Lamont et al., 2018). When sufficient fermentable carbohydrates are overexposed, the balance between commensals and pathogens is disrupted. Large amounts of glucans, fructans (synthesize EPS), and lactic acid are produced by pathogens, which induce the pH of the local microenvironment to fall below 5.5 (Takahashi and Nyvad, 2011; Xiao et al., 2012; Bowen et al., 2018). In turn, this acidic environment provides a conditional cariogenic nest and further promotes microbial shifts toward pathogens with acid-resistant capability (Takahashi and Nyvad, 2011; Marsh and Zaura, 2017; Bowen et al., 2018). This favors the demineralization and disintegration of local mineralized tooth tissue and the onset of caries (Pitts et al., 2017). Therefore, this sort of anti-caries strategy that interferes with the acidic environment and inhibits the proliferation and metabolism of caries-related pathogens has been sought after by researchers to attempt to explore a way for the conundrum. Many novel pH-sensitivity biological materials have been applied to advance the feasibility of this strategy (Horev et al., 2015; Naha et al., 2019).

The proton pump FoF1-ATPase (also named H^+^-ATPase) is a critical action enzyme involved in the acid-resistant mechanism for microbial (Liu et al., 2015). It is a ubiquitous and evolutionarily strong conserved membrane-bound macromolecular enzyme among prokaryotes and eukaryotes, it acts as the powerhouse of cell, and its transmembrane domain (Fo complex, subunit *c*) mediates the proton transport to resist low pH environment (Fillingame and Dmitriev, 2002). Hence, the FoF1-ATPase is a vital target for drugs that treat acid-related diseases (Spugnini and Fais, 2017; Abe et al., 2018), such as omeprazole targeting ATPase for gastric ulcer disease (Sachs, 1984), bedaquiline targeting Fo ring of *Mycobacterium tuberculosis* for tuberculosis (Preiss et al., 2015), and some proton pump inhibitions for cancer (Spugnini and Fais, 2017). However, dental caries is also an acid-related disease, and few studies have targeted H^+^-ATPase of caries-related microorganisms to explore caries prevention. For human non-toxicity and safety concerns, a key factor to consider for screening new antibacterial drugs targeting the H^+^-ATPase domain is the lack of eukaryotic homolog of the target.

During our continuous search for potential anti-caries agent, we identified bedaquiline, an antibiotic that was initially developed to specifically inhibit the mycobacterial ATP-synthase (Preiss et al., 2015) and does not recruit ATP synthesis-related toxicity in mammalian cells (Haagsma et al., 2009; Narang et al., 2019). The strong inhibitory effect of bedaquiline on *Streptococcus mutans* confirmed in preliminary experiments led us to propose that bedaquiline could be repurposed as a novel anti-caries agent, for the targeting H^+^-ATPase. Therefore, here, the *in vitro* antibacterial activity of bedaquiline against *S. mutans* and multispecies genera (planktonic and biofilm) was investigated.

## Materials and Methods

### Bacteria Inoculation and Biofilm Formation

*S. mutans* UA159 (ATCC10449) provided by Professor Mingwen Fan (Wuhan University, Wuhan, China), *Streptococcus sanguinis*, and *Streptococcus salivarius* isolated by Qiang Feng group (Shandong University, Jinan, China) were routinely inoculated in brain–heart infusion broth (BHI; BD Difco, United States) at an 37°C anaerobic incubator (90% N_2_, 5% CO_2_, 5% H_2_; Whitley DG250 anaerobic workstation, United Kingdom). The prepared bacterial suspension [10^8^ colony-forming units (CFUs)/ml, logarithmic phase] was obtained by transferring and incubating the overnight culture products of bacteria at a ratio of 1:40 for 3–6 h (*S. mutans* for 4 h; *S. sanguinis* for 6 h; *S. salivarius* for 3 h). For biofilm formation, the prepared bacterial suspension was inoculated into fresh BHI (1:100) with 1% (wt/vol) sucrose in multi-well cell (96-well and 24-well) culture plates for 24 h.

### Minimum Inhibitory Concentration (MIC) and IC_50_ Concentration Assay

The MIC determination for bedaquiline (purchased from MedChemExpress) against planktonic *S. mutans* was conducted using the microdilution method in accordance with the Clinical Laboratory Standards Institute (CLSI) guideline (Pfaller and Diekema, 2012), with some modifications as described below. Bedaquiline powder was dissolved in DMSO to prepare a 10 mg/ml storage solution and then serially diluted in BHI medium to obtain 20, 10, 5, 2.5, 1.25, 0.625, and 0.3 mg/L working solution. One hundred microliters of serially diluted working solution with various concentrations of bedaquiline was mixed with 100 μl of the prepared bacterial suspension (10^7^ CFU) in a 96-well plate. BHI with corresponding concentrations of DMSO were used as control and three parallel samples were set at each concentration. The plates were incubated anaerobically at 37°C for 24 h. The optical density value of 600 nm (OD_600 nm_) was detected with a microplate reader (SPECTROstar Nano, BMG, Germany) to evaluate the growth status of bacteria.

The IC_50_ determination for bedaquiline against planktonic *S. mutans* was similar to the above procedure. Each well contained 1, 1.5, 2, 2.5, 3, 3.5, 4, 4.5, and 5 mg/L bedaquiline and 10^7^ CFU bacterial suspension for incubation. The procedure was also conducted on the control group and parallel samples. Meanwhile, the simplification procedure was set and performed to determine the MIC and IC_50_ of *S. sanguinis* and *S. salivarius* (only dosage concentrations of 1, 2, 2.5, 3, and 4 mg/L were tested). The MIC was defined as the lowest test concentration that substantially inhibited bacteria growth in the medium. The IC_50_ was defined as the concentration at which half visible bacteria grew in the medium.

Furthermore, the antibacterial effect of bedaquiline under different pH milieus (pH 4–7) was also investigated. Hydrochloric acid was used to prepare the pH of the BHI; the prepared bacterial suspension was mixed with acidic BHI incorporating 1–3 mg/L of bedaquiline (bacterial suspension: BHI medium = 1:100), and the OD_600 nm_ value was tested after 24 h of anaerobic culture at 37°C.

### CFU Counts and Drop Assay

For CFU counts, bacterial suspension treated 0, 5, 10, 30 min, and 1, 2, 4, 6, and 8 h with bedaquiline in pH 5 BHI broth was serially diluted in PBS and plated 100 μl on BHI agar for incubation and CFU counts. In parallel, 10 μl of bacterial suspension at various time points was dropped on pH 7 BHI agar to visually detect the biomass.

### Scanning Electron Microscopy (SEM) Imaging

For SEM imaging, 24-h incubated *S. mutans* and multispecies biofilms on coverslip disks in 24-well plate were used for processing and imaging (Zhang et al., 2015). Briefly, the biofilms were washed twice with PBS, fixed with 2.5% glutaraldehyde overnight, serially dehydrated with ethanol, gradient frozen, and dried for 12 h in freeze-dryers (Martin Christ, Germany). Then, the samples were sputter-coated with gold for SEM imaging.

### Lactic Acid Measurement and pH Measurement

The biofilms for lactic acid measurement were incubated in 24-well plates with 1 ml of pH 5 BHI broth (Zhang et al., 2015). Follow the instructions (LA Assay Kit, Solarbio, China) to monitor the lactic acid production at OD_340 *nm*_. The supernatant of biofilm incubated in pH 5 BHI broth for 2, 4, 6, 8, and 16 h was used for pH measurement by Starter (Starter 3100, United States).

Furthermore, to evaluate the effect of bedaquiline on the lactic acid production of mature *S. mutans* biofilm (obtained by culturing in pH 7 BHI for 16 h), the shock assay was designed and performed. Based on the CFU count results of the antibacterial effect of bedaquiline with time gradient, the mature *S. mutans* biofilm was shocked for 2 h in pH 5 BHI with the incorporation of 2.5, 4, and 10 mg/L of bedaquiline, and then the lactic acid production of the shocked biofilms was detected as above. In parallel, pH changes of the shocked biofilms were recorded.

### Polysaccharide Measurement

The biofilms were incubated in 24-well plates with 1 ml of pH 5 BHI broth. The water-insoluble extracellular polysaccharide of biofilms was determined by the anthrone method, the procedure referred to in Koo et al. (2003). Briefly, the cell pellet of biofilm was resuspended and washed thrice in sterile PBS and then resuspended using 4 ml of 0.4 M NaOH and centrifuged at 5,000 rpm for 3 min, and 200 μl of supernatant was mixed with 600 μl of freshly prepared anthrone–sulfuric acid solution [1 g/L (80% sulfuric acid)]. Simultaneously, 200 μl of freshly prepared dextran T 500 standard with various concentrations (0, 0.005, 0.01, 0.02, …, 0.09, and 0.1 mg/ml) were also mixed with 600 μl of freshly prepared anthrone–sulfuric acid solution for comparison. Put samples and standards in a 95°C dry bath for 6 min and immediately transfer them to the ice box for 15 min. Pipette 200 μl into a 96-well plate and detect the absorbance value of OD_625nm_. Three parallel groups were set for each group. In addition, polysaccharide production assay of the shocked biofilms was also performed.

### Live/Dead Bacteria Imaging

For live/dead imaging (Zhang et al., 2015), mature biofilms shocked in pH 5 BHI containing 2.5–10 mg/L of bedaquiline were stained following the manufacturer’s instruction (Live/Dead Baclight^TM^ Bacterial viability kits, Invitrogen^TM^, United States). Briefly, the biofilms were stained with SYTO 9 for 15 min and then propidium iodide for 3 min. The labeled biofilms were imaged with a fluorescence microscope (Leica DMi8, Wetzlar, Germany) equipped with fully consistent exposure value (147), magnification (10×), and other parameters.

### CCK8 Assay

The periodontal ligament stem cells (PDLSCs, P5) isolated and identified by our research group were activated and expanded for 48 h (5 × 10^5^ live cells/ml), plating 3,000 cells/well in a 96-well plate. After monolayer culture overnight, the medium (DMEM + 10% FBS) containing different concentrations of bedaquiline (1–10 mg/L) and DMSO was replaced; culture is continued for 24, 48, and 72 h; and then the effect of bedaquiline on cell proliferation (OD_450 *nm*_) was tested with Cell counting kit-8 (CCK8, DOJINDO, Japan). Five parallel groups were set for each group (Ibrahim et al., 2020).

### H^+^-ATPase Activity Assay

Freshly activated *S. mutans* solution in stable phase (100 ml, 10^10^ CFU/ml) was prepared, and then the total protein was extracted by lysing the cell pellet with lysate and lysozyme. Protein concentration was obtained using the Bicinchoninic Acid Kit (BAC, Sigma-Aldrich, United States). An equal amount of 50 μg protein was dispensed and treated with 56–139 mg/L of bedaquiline or DMSO at 37°C for 30 min (negative controls with a corresponding concentration of bedaquiline were also set), and then the activity of H^+^-ATPase at 1, 10, and 30 min after substrate incorporation was detected with the H^+^-ATPase assay kit (GMS50244.3, Genmed Scientifics Inc., United States, OD_340 *nm*_) (Iwamoto et al., 1991).

### Statistical Analysis

IBM SPSS Statistics version 17.0 was used to perform the statistical analysis and graphs were drawn using GraphPad. All experiments were independently repeated at least three times. One-way analysis of variance (ANOVA) and Tukey’s test were performed to detect the significant effects of multiple groups. The *t*-test was performed for two groups. Differences were considered significant when *P* < 0.05.

## Results

### Determination of the MIC and IC_50_ of Bedaquiline Against *S. mutans*

**FIGURE 1:**
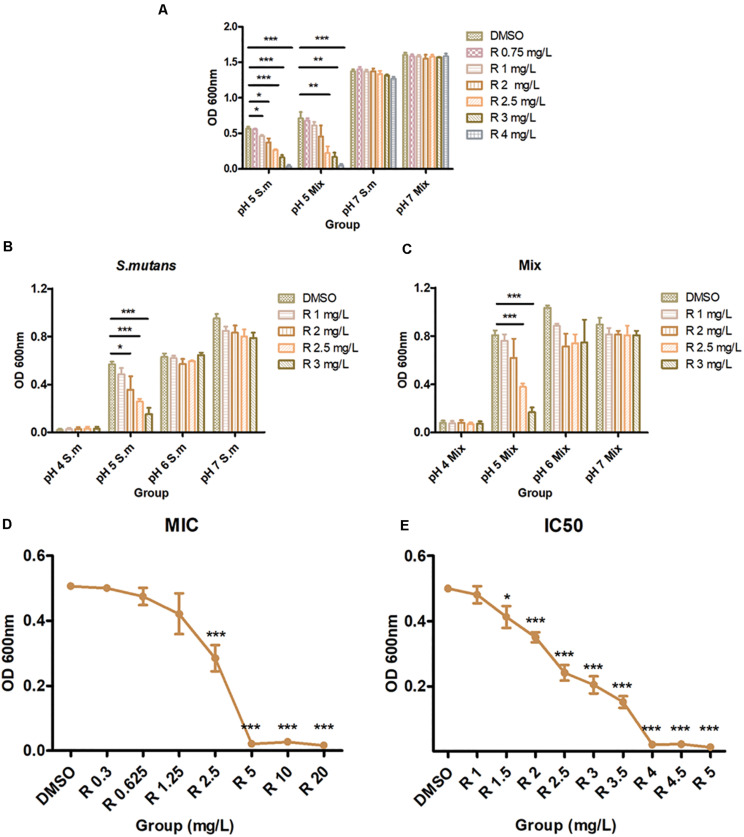
Antibacterial properties of bedaquiline treatment. **(A)** In pH 5 and 7 BHI, the antibacterial effect of 0.75–4 mg/L of bedaquiline and DMSO against planktonic *S. mutans* and Mix (*S. mutans, S. sanguinis*, and *S. salivarius*). **(B)** In pH 4–7 BHI, the antibacterial effect of 1–3 mg/L of bedaquiline and DMSO against planktonic *S. mutans*. **(C)** In pH 4–7 BHI, the antibacterial effect of 1–3 mg/L of bedaquiline and DMSO against planktonic Mix. **(D)** The MIC assay of bedaquiline against planktonic *S. mutans* in pH 5 BHI. **(E)** The IC_50_ assay of bedaquiline against planktonic *S. mutans* in pH 5 BHI. *, **, and *** indicate statistically significant differences at *p* < 0.05, *p* < 0.01, and *p* < 0.001, respectively. Error bars are standard deviations.

The activity of bedaquiline against *S. mutans* and multispecies suspension are summarized in Figure 1. In pH 7 BHI, 0.75–4 mg/L of bedaquiline (Figure 1A) and even 10 mg/L (data not shown) did not exhibit bacteriostatic effects, but exhibited excellent bacteriostatic effects in pH 5 BHI (Figure 1A). The bactericidal effect test at pH 4–7 BHI also evidenced that bedaquiline performed excellent bacteriostatic effect on *S. mutans* and Mix at pH 5 (Figures 1B,C). Figures 1D,E show the results of MIC and IC_50_ assay; bedaquiline demonstrated an MIC of 4 mg/L, with an IC_50_ of 2.5 mg/L against planktonic *S. mutans*. For *S. sanguinis*, MIC = 4 mg/L and IC_50_ = 2 mg/L. For *S. salivarius*, MIC = 4 mg/L and 2 mg/L < IC_50_ < 2.5 mg/L (Supplementary Figures S1, S2). Similar inhibitory concentrations were also presented in the assay with mixed multiple species (Figure 1A).

**FIGURE 2:**
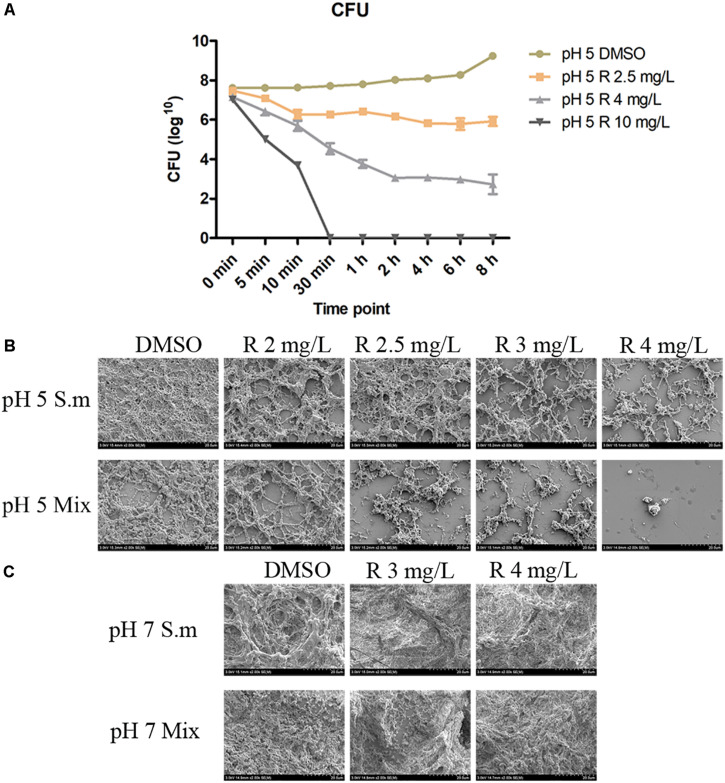
The CFU count **(A)** of 2.5–10 mg/L of bedaquiline and DMSO against planktonic *S. mutans* in pH 5 BHI. Error bars are standard deviations. **(B,C)** Are anti-biofilm properties of bedaquiline (SEM). **(B)** In pH 5 BHI, anti-biofilm generation effect of 2–4 mg/L of bedaquiline and DMSO against *S. mutans* and Mix. **(C)** In pH 7 BHI, anti-biofilm generation effect of 3 and 4 mg/L of bedaquiline and DMSO against *S. mutans* and Mix.

CFU count results showed that *S. mutans* grows and proliferates slowly in the first 6 h in the pH 5 BHI and remains in the 10^8^ level (Figure 2A). After the incorporation of bedaquiline, the 2.5 mg/L group can reduce the bacteria amount to 10^6^ levels within 10 min and stay at this level; the 4 mg/L group continued to decrease to 10^3^ level in the first 2 h, reaching its limit of inhibition, but the inhibitory effect continued until 8 h; the 10 mg/L group achieved complete inhibition of bacteria within 30 min. The similar results of drop assay (Supplementary Figure S3) further provided an extra visual evidence for the dose-dependent and time-dependent effects of bedaquiline.

### Inhibition of Biofilm Formation by Bedaquiline

The SEM images showed that the acidic environment itself affected biofilm formation (Figures 2B,C), and the incorporation of bedaquiline further inhibited the development of *S. mutans* and Mix biofilms to varying degrees (Figure 2B), but had no obvious effect on the formation of biofilms in a neutral environment (Figure 2C). The density of *S. mutans* biofilm formation with 2, 2.5, 3, and 4 mg/L of bedaquiline in pH 5 BHI gradually reduced in a dose-dependent manner. More intense inhibition of bedaquiline was shown in the Mix biofilm group.

### Evaluation of the Lactic Acid Production of *S. mutans* Biofilm With Bedaquiline

**FIGURE 3:**
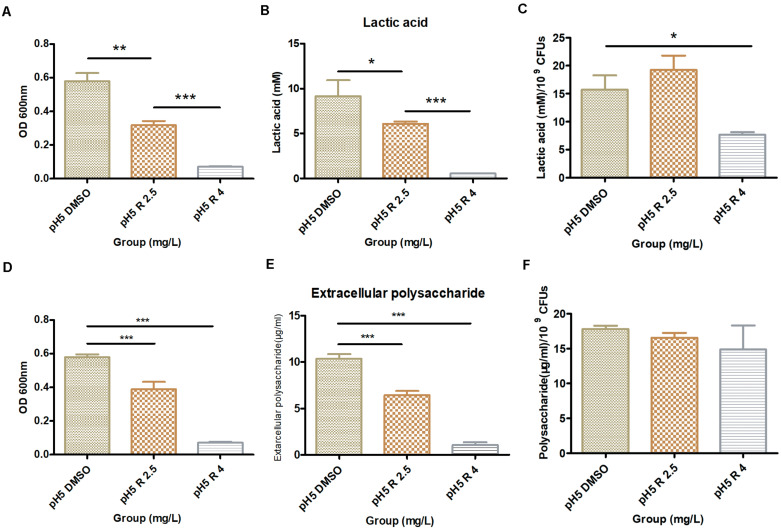
Effect of 2.5 and 4.0 mg/L of bedaquiline and DMSO on lactic acid production and water-insoluble extracellular polysaccharide production within *S. mutans* biofilm in pH 5 BHI. **(A)** OD_600 nm_ value of *S. mutans* biofilm used for lactic acid detection. **(B)** Lactic acid production detection in different groups. **(C)** Lactic acid production/10^9^ CFUs of different groups. **(D)** OD_600 nm_ value of *S. mutans* biofilm used for water-insoluble extracellular polysaccharide detection. **(E)** Extracellular polysaccharide detection in different groups. **(F)** Extracellular polysaccharide production/10^9^ CFUs of different groups. *, **, and *** indicate statistically significant differences at *p* < 0.05, *p* < 0.01, and *p* < 0.001, respectively. Error bars are standard deviations.

Compared to the control group, *S. mutans* biofilms with 2.5 and 4 mg/L of bedaquiline in pH 5 BHI significantly reduced the lactic acid production (*P* < 0.05; Figure 3B). However, the incorporation of bedaquiline also significantly reduced the amounts of bacteria in the biofilm (Figure 3A), so the ratio of lactic acid production to OD_600 nm_ in the 2.5 mg/L group was not statistically different from the control group (Figure 3C).

The results of lactic acid production of the shocked biofilms showed that no significant differences were statistically obtained when the 2.5 and 4 mg/L groups were compared with the control group, even though the lactic acid production slightly decreased with increasing inhibitor concentration (Supplementary Figure S4).

### Evaluation of the Water-Insoluble Polysaccharide Production of *S. mutans* Biofilm With Bedaquiline

The results of the water-insoluble extracellular polysaccharide production were similar to those of lactic acid. The polysaccharides in the 2.5 and 4 mg/L group decreased considerably (*P* < 0.05, Figure 3E), and the amounts of bacteria that make up the biofilm (OD_600nm_) were also significantly inhibited (Figure 3D), which kept the ratio of the polysaccharide production to OD_600nm_ at a relatively conforming level (*P* > 0.05, Figure 3F). The yield of polysaccharides in shocked biofilms also showed a tendency to decrease slightly with increasing inhibitor concentration, but no statistical difference was shown (Supplementary Figure S5).

### pH Dynamic Changes of the Medium of the Planktonic Bacteria and Shocked Biofilm After the Incorporation of Bedaquiline

**FIGURE 4:**
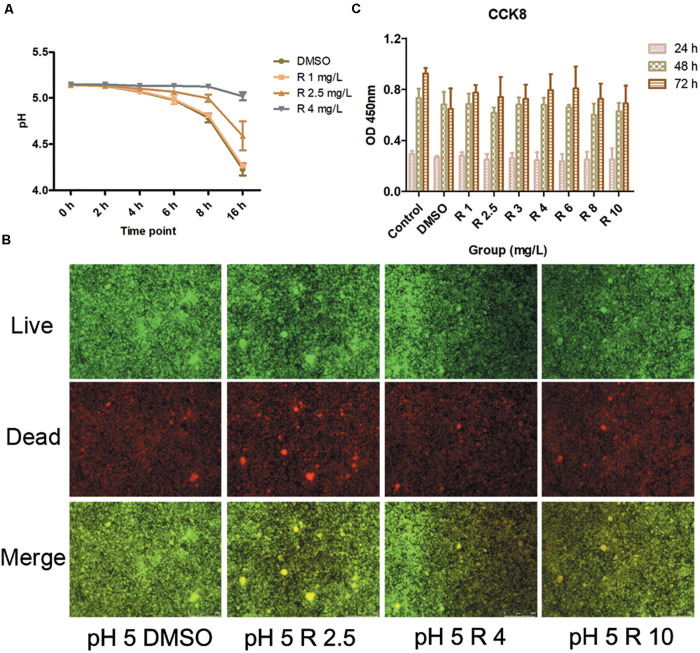
**(A)** pH detection at 0–16 h culturation after the incorporation of 1–4 mg/L of bedaquiline and DMSO. **(B)** Live/dead bacteria stain imaging of *S. mutans* biofilm with 2.5, 4, and 10 μM of bedaquiline and DMSO treatment. **(C)** Cytotoxic effects of 1–10 mg/L of bedaquiline and DMSO on periodontal ligament stem cells at 24, 48, and 72 h.

The incorporation of bedaquiline retarded the pH drops (Figure 4A). The pH in the control group and the 1 mg/L group gradually dropped from 5.14 ± 0.01 to 4.25 ± 0.05 in 16 h, and the pH in the 2.5 mg/L group dropped slightly slowly to 4.59 ± 0.16 vs. that in the control group (*P* = 0.02). The pH in the 4 mg/L group dropped most slowly, staying at about 5.14 in 16 h. Moreover, the pH measurement of the shocked biofilms showed that no significant differences were obtained between the 2.5 and 4 mg/L group and the control group (data not shown).

### Evaluation of the Live/Dead Bacteria Ratio in Shocked *S. mutans* Biofilm

Under a fluorescence microscope, green indicates live bacteria, red indicates dead bacteria, and yellow turned darker in the merge image when the proportion of dead bacteria was higher. As the concentration of incorporated bedaquiline increased, the area and depth of the yellow area increased (Figure 4B). However, even after treatment with the 10 mg/L group with complete bacteriostatic ability, the yellow brightness in the merge was only slightly dimmed.

### Cytotoxicity Assessment of Bedaquiline

Cell viability in bedaquiline groups (1–10 mg/L) at 24and 48 h were not significantly different from the DMSO group (0.1%) and the control group (*P* > 0.05, Figure 4C). At 72 h, cell viability of bedaquiline groups and the DMSO group fluctuated to some extent, while statistics showed that there was no difference between the groups. However, the cell viability in the DMSO group at 72 h was reduced compared with the control group (*P* = 0.055), which indicated that 0.1% DMSO would bring some cytotoxicity with time, but bedaquiline below 10 mg/L did not show cytotoxicity.

### Evaluation of the Inhibition of H^+^-ATPase Activity by Bedaquiline

**FIGURE 5:**
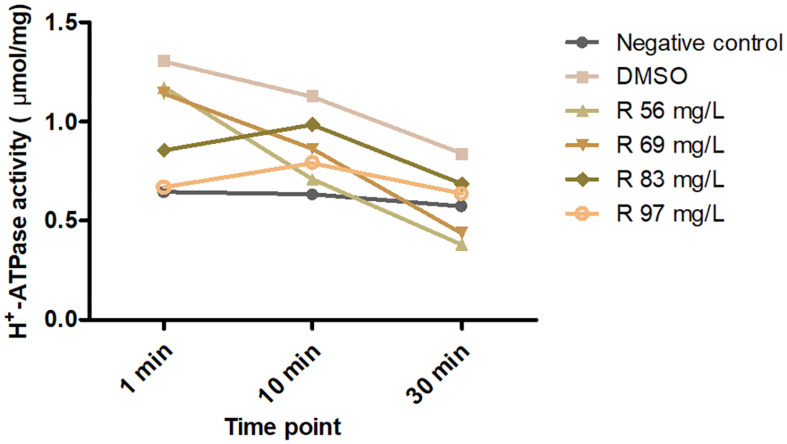
Effects of 56–97 mg/L of bedaquiline and DMSO on H^+^-ATPase activity of *S. mutans* at 0, 10, and 30 min.

A total of 2.5 ml of 1 μg/μl total protein was extracted. The ≤56 mg/L of bedaquiline groups was the same as the DMSO group, and H^+^-ATPase was gradually consumed by the substrate within 30 min, showing a slow downward trend (Figure 5). In the 69, 83, and 97 mg/L groups, H^+^-ATPase activity at 0 min was inhibited to various extent, and as the concentration increased, there was a gradual downward trend of the curve within 30 min. In the ≥97 mg/L groups, the curves were almost a horizontal line, indicating that the activity of H^+^-ATPase had been completely inhibited, and there was no H^+^-ATPase that could react with the substrate.

## Discussion

Here, we determined the antibacterial effects of bedaquiline against *S. mutans* and multiple species in acidic milieus, and its inhibitory effect on H^+^-ATPase protein *in vitro*. These results evidenced that the way to inhibit H^+^-ATPase, an acid-resistant functional protein, to resist caries-related microorganisms in an acidic caries environment is available. However, considering the use of bedaquiline is FDA approved for the treatment of MDR *M. tuberculosis*, the likelihood of antibiotic resistance among *M. tuberculosis* will increase if it is used directly as an anti-caries agent (Diacon et al., 2014). Therefore, subsequent research mainly focused on improving the feasibility of topical application of drugs by modifying bedaquiline and encapsulating it with nanomaterials to reduce its impact on the whole body.

Caries development is a consequence of dietary sugar-driven biofilm accumulation and localized acidification caused by deleterious microbes (Takahashi and Nyvad, 2011; Colombo and Tanner, 2019); this increased acidification is accompanied by the reduction in the levels and metabolic activity of beneficial bacteria, which preferentially grow at neutral pH (Teng et al., 2015; Johansson et al., 2016). At present, many antibacterial studies against dental caries mainly focus on their bactericidal effect in neutral microenvironment (Saputo et al., 2018; Qi et al., 2019; Ibrahim et al., 2020). Obviously, many innocent beneficial flora will be mutilated simultaneously by this bactericidal effect, destroying the local flora balance. A key point to remember is that those bacteria with acid-resistant capability (that is, caries-related bacteria) left behind by the conditional screening in acidic microenvironment are those we want to fight. Therefore, several novel pH-sensitive biological materials that load antibacterial drugs (Horev et al., 2015; Naha et al., 2019), antibacterial peptides with pH sensitivity (Zhang et al., 2015), and precision-guided antimicrobial peptides (Guo et al., 2015) have emerged to assist to overcome this. Here, in acidic milieus, bedaquiline in the low micromolar range [2.5 mg/L (IC_50_), 4 mg/L (MIC); Figure 1] potently inhibited the proliferation of *S. mutans* and other caries-related bacteria, yet the antibacterial effect is not demonstrated in neutral milieus. It is favorable to maintain local biological balance by protecting healthy microflora and targeting only those caries-related microbiomes that are resistant to an acidic environment. Hence, bedaquiline is worth further exploration to assist in preventing dental caries.

The sucrose metabolism of microorganisms in the biofilm is closely related to the pH value in the local microenvironment (Xiao et al., 2012; Flemming et al., 2016; Hwang et al., 2016; Bowen et al., 2018). This extracellular matrix, including exopolysaccharides, glycoproteins, and lipoteichoic acid, has been increasingly recognized as essential for the cariogenic properties involved in surface adhesion, social interactions, and antimicrobial tolerance (Flemming et al., 2016; Bowen et al., 2018). Therefore, detecting the effect of antibacterial agent on microbial sucrose metabolism has been increasingly valued to assess its anti-caries potential, and commonly used indicators include lactic acid production, water-insoluble extracellular polysaccharide production, and pH monitoring (Zhang et al., 2015; Henley-Smith et al., 2018; Yu et al., 2019; Chen et al., 2020; Ibrahim et al., 2020). Consistent with many previous studies (Henley-Smith et al., 2018; Yu et al., 2019; Chen et al., 2020; Ibrahim et al., 2020), the incorporation of antibacterial agent, including bedaquiline, noticeably reduced both the lactic acid production and total water-insoluble extracellular polysaccharides compared to the control group (Figure 3); the drop in pH 5 was also significantly reduced (Supplementary Figure S3). However, the initial sugar production of pH 5 BHI in the control group in the present study is lower than that of pH 7 BHI in other research (Zhang et al., 2015; Yu et al., 2019), which may largely be related to the inhibitory effect of the acidic environment on the amount of bacteria and bacterial metabolism. In addition, the reduction extent in sugar production in the bedaquiline group is also not as great as in other studies, even in a similar situation where half of the bacterial biomass was suppressed (Zhang et al., 2015). Here, supplementary evidence shows that there is no difference in the proportion of sugar production/OD_600 nm_ between the bedaquiline group and the control group. We speculate that the inhibitory effect of bedaquiline on sugar production may be mainly obtained by reducing the biomass of bacteria, but has no effect on sugar metabolism itself.

In addition, the present study shows that once the mature biofilm has been constructed, even the shock treatment with 10 mg/L of bedaquiline only causes the death of a small number of bacteria on the surface of the biofilm (Figure 4B), and there is no obvious change in the sugar yield and pH of the biofilm (Supplementary Figures S4, S5). This is consistent with the fact that biofilm itself is a complex and united small community; it is difficult to be attacked and influenced by some foreign drug molecules or agents, which is also a mechanism for its self-protection (Flemming et al., 2016; Bowen et al., 2018). This result also indicates that when bedaquiline is used as an anti-caries agent in the future, it should be directly applied on tooth surface or active lesion, or with the assist of novel biological nanomaterials to increase the permeability of bedaquiline to the biofilm (Naha et al., 2019).

The Fo protein of H^+^-ATPase is utilized to pump out H^+^ from cells, thereby maintaining the pH homeostasis and protecting cells from damage induced by acidic milieus. This mechanism is shared by various bacteria. Kuhnert’s group (Kuhnert et al., 2004) found the transcriptionally upregulated H^+^-ATPase in *S. mutans* confronted with a low pH environment, which confirms its critical role in acid resistance. Bedaquiline, an inhibitor of mycobacterial F1Fo-ATP synthase that binds to the enzyme’s oligomeric *c* subunit of Fo protein (Andries et al., 2005; Koul et al., 2007; Preiss et al., 2015), has been approved by FDA for the treatment of drug-resistant *M. tuberculosis* disease (Diacon et al., 2009; Jones, 2013). Haagsma’s group showed that ATP synthase isolated from human, mouse, and bovine mitochondria displayed extremely lower sensitivity for bedaquiline compared to that of mycobacterial ATP synthase (Haagsma et al., 2009), which indicates that bedaquiline is able to selectively inhibit ATP synthase in bacteria but not in mitochondria of normal cells (Fiorillo et al., 2016). This property contributes to its clinical usage as antibacterial agent due to toxicity issues and fatality concerns (Ferlazzo et al., 2018; Narang et al., 2019). Therefore, the potent inhibitory effect of bedaquiline on oral microorganisms and the non-toxic effect on PDLSCs in the present study laid a solid foundation for bedaquiline as a novel anti-caries agent.

The precise interaction of bedaquiline with the c-ring of Fo rotors and its mechanism of action have been explored and expounded by Preiss’ group in mycobacterial ATP synthases (Preiss et al., 2015). They confirmed that the bedaquiline specifically interacts with nine residues on c-rings of *Mycobacterium phlei* (Gly^62^, Leu^63^, Glu^65^, Ala^66^, Ala^67^, Tyr^68^, Phe^69^, Ile^70^, and Leu^72^) using X-ray crystallographic study. Among them, the glutamate residue with the strictly conserved bacterial ATP synthases (Glu^65^ in *M. phlei*, Glu^53^ in *S. mutans*) plays an essential role in binding and translocating H^+^ and other ions during the ion translocation process (Pogoryelov et al., 2010; Hakulinen et al., 2012). Here, the inhibitory effect of bedaquiline on H^+^-ATPase in *S. mutans* is confirmed (Figure 5); the conserved glutamate residue is presumed to be a key binding site for bedaquiline, but other potential binding sites in *S. mutans* need to be further studied.

Here, the inhibition effect of the H^+^-ATPase inhibitor on caries-related pathogens in an acidic environment paves the way for the development of a novel anti-caries strategy option. However, since this study was conducted *in vitro*, and only a limited number of species were applied to research, it failed to accurately simulate the natural oral environment. Further *in vivo* research is needed to provide higher levels of evidence. Moreover, the inhibition effect of bedaquiline against *S. mutans* will definitely cause bacterial resistance over time, which is an issue that requires our continuous attention and cannot be ignored.

## Conclusion

In conclusion, bedaquiline is efficient in inhibiting the proliferation and biofilm generation of *S. mutans* and other oral pathogens in an acidic environment. Its high targeting property and non-cytotoxicity promote its clinical application potential in preventing caries. Further mechanism exploration and drug modification are warranted to advance its feasibility.

## Data Availability Statement

The original contributions presented in the study are included in the article/Supplementary Material, further inquiries can be directed to the corresponding author/s.

## Author Contributions

DZ, XX, and EL conceived this study. MZ, WY, SZ, and BZ conducted the experiments. MZ and EL analyzed the data and wrote the manuscript. All authors contributed to the article and approved the submitted version.

## Conflict of Interest

The authors declare that the research was conducted in the absence of any commercial or financial relationships that could be construed as a potential conflict of interest.

## References

[B1] Abe K. Irie K. Nakanishi H. Suzuki H. Fujiyoshi Y. (2018). Crystal structures of the gastric proton pump. *Nature* 556 214–218. 10.1038/s41586-018-0003-8 29618813

[B2] Andries K. Verhasselt P. Guillemont J. Göhlmann H. W. Neefs J. M. Winkler H. (2005). A diarylquinoline drug active on the ATP synthase of Mycobacterium tuberculosis. *Science* 307 223–227. 10.1126/science.1106753 15591164

[B3] Benoit D. S. W. Sims K. R. Fraser D. (2019). Nanoparticles for oral biofilm treatments. *ACS Nano.* 13 4869–4875. 10.1021/acsnano.9b02816 31033283 PMC6707515

[B4] Bowen W. H. Burne R. A. Wu H. Koo H. (2018). Oral biofilms: pathogens, matrix, and polymicrobial interactions in microenvironments. *Trends Microbiol.* 26 229–242. 10.1016/j.tim.2017.09.008 29097091 PMC5834367

[B5] Chen H. Tang Y. Weir M. D. Gao J. Imazato S. Oates T. W. (2020). Effects of S. mutans gene-modification and antibacterial monomer dimethylaminohexadecyl methacrylate on biofilm growth and acid production. *Dent. Mater.* 36 296–309. 10.1016/j.dental.2019.12.001 31839202

[B6] Colombo A. P. V. Tanner A. C. R. (2019). The role of bacterial biofilms in dental caries and periodontal and peri-implant diseases: a historical perspective. *J. Dent. Res.* 98 373–385. 10.1177/0022034519830686 30890060

[B7] Diacon A. H. Pym A. Grobusch M. Patientia R. Rustomjee R. Page-Shipp L. (2009). The diarylquinoline TMC207 for multidrug-resistant tuberculosis. *New Engl. J. Med.* 360 2397–2405. 10.1056/nejmoa0808427 19494215

[B8] Diacon A. H. Pym A. Grobusch M. P. de los Rios J. M. Gotuzzo E. Vasilyeva I. (2014). Multidrug-resistant tuberculosis and culture conversion with bedaquiline. *New Engl. J. Med.* 371 723–732. 25140958 10.1056/NEJMoa1313865

[B9] Ferlazzo G. Mohr E. Laxmeshwar C. Hewison C. Hughes J. Jonckheere S. (2018). Early safety and efficacy of the combination of bedaquiline and delamanid for the treatment of patients with drug-resistant tuberculosis in Armenia, India, and South Africa: a retrospective cohort study. *Lancet Infect. Dis.* 18 536–544. 10.1016/s1473-3099(18)30100-2 29452942

[B10] Fillingame R. H. Dmitriev O. Y. (2002). Structural model of the transmembrane Fo rotary sector of H+-transporting ATP synthase derived by solution NMR and intersubunit cross-linking in situ. *Biochim. Biophys. Acta.* 1565 232–245. 10.1016/s0005-2736(02)00572-2 12409198

[B11] Fiorillo M. Lamb R. Tanowitz H. B. Cappello A. R. Martinez-Outschoorn U. E. Sotgia F. (2016). Bedaquiline, an FDA-approved antibiotic, inhibits mitochondrial function and potently blocks the proliferative expansion of stem-like cancer cells (CSCs). *Aging* 8 1593–1607. 10.18632/aging.100983 27344270 PMC5032685

[B12] Flemming H. C. Wingender J. Szewzyk U. Steinberg P. Rice S. A. Kjelleberg S. (2016). Biofilms: an emergent form of bacterial life. *Nat. Rev. Microbiol.* 14 563–575. 10.1038/nrmicro.2016.94 27510863

[B13] Gao L. Liu Y. Kim D. Li Y. Hwang G. Naha P. C. (2016). Nanocatalysts promote *Streptococcus mutans* biofilm matrix degradation and enhance bacterial killing to suppress dental caries in vivo. *Biomaterials* 101 272–284. 10.1016/j.biomaterials.2016.05.051 27294544 PMC4949957

[B14] Guo L. McLean J. S. Yang Y. Eckert R. Kaplan C. W. Kyme P. (2015). Precision-guided antimicrobial peptide as a targeted modulator of human microbial ecology. *PNAS* 112 7569–7574. 10.1073/pnas.1506207112 26034276 PMC4475959

[B15] Haagsma A. C. Abdillahi-Ibrahim R. Wagner M. J. Krab K. Vergauwen K. Guillemont J. (2009). Selectivity of TMC207 towards mycobacterial ATP synthase compared with that towards the eukaryotic homologue. *Antimicrob. Agents Chemother.* 53 1290–1292. 10.1128/aac.01393-08 19075053 PMC2650532

[B16] Hakulinen J. K. Klyszejko A. L. Hoffmann J. Eckhardt-Strelau L. Brutschy B. Vonck J. (2012). Structural study on the architecture of the bacterial ATP synthase Fo motor. *PNAS* 109 E2050–E2056. 22736796 10.1073/pnas.1203971109 PMC3409797

[B17] Henley-Smith C. J. Botha F. S. Hussein A. A. Nkomo M. Meyer D. Lall N. (2018). Biological activities of *Heteropyxis natalensis* against micro-organisms involved in oral infections. *Front. Pharmacol.* 9:291. 10.3389/fphar.2018.00291 29692723 PMC5903190

[B18] Horev B. Klein M. I. Hwang G. Li Y. Kim D. Koo H. (2015). pH-activated nanoparticles for controlled topical delivery of farnesol to disrupt oral biofilm virulence. *ACS Nano.* 9 2390–2404. 10.1021/nn507170s 25661192 PMC4395463

[B19] Hwang G. Liu Y. Kim D. Sun V. Aviles-Reyes A. Kajfasz J. K. (2016). Simultaneous spatiotemporal mapping of in situ pH and bacterial activity within an intact 3D microcolony structure. *Sci. Rep.* 6:32841. 10.1038/srep32841 PMC5015094 27604325

[B20] Ibrahim M. S. Garcia I. M. Vila T. Balhaddad A. A. Collares F. M. Weir M. D. (2020). Multifunctional antibacterial dental sealants suppress biofilms derived from children at high risk of caries. *Biomater. Sci.* 8 3472–3484. 10.1039/d0bm00370k 32432287

[B21] Iwamoto A. Omote H. Hanada H. Tomioka N. Itai A. Maeda M. (1991). Mutations in Ser174 and the glycine-rich sequence (Gly149, Gly150, and Thr156) in the beta subunit of *Escherichia coli* H(+)-ATPase. *J. Biol. Chem.* 266 16350–16355. 10.1016/s0021-9258(18)55304-7 1832155

[B22] Johansson I. Witkowska E. Kaveh B. Lif Holgerson P. Tanner A. C. (2016). The microbiome in populations with a low and high prevalence of caries. *J. Dent. Res.* 95 80–86. 10.1177/0022034515609554 26442950 PMC4700664

[B23] Jones D. (2013). Tuberculosis success. *Nat. Rev. Drug Discovery* 12 175–176. 23449293 10.1038/nrd3957

[B24] Kassebaum N. J. Smith A. G. C. Bernabé E. Fleming T. D. Reynolds A. E. Vos T. (2017). Global, regional, and national incidence, prevalence, and years lived with disability for 328 diseases and injuries for 195 countries, 1990-2016: a systematic analysis for the Global Burden of Disease Study 2016. *Lancet (London, Engl.)* 390 1211–1259. 10.1016/S0140-6736(17)32154-2 PMC5605509 28919117

[B25] Koo H. Hayacibara M. F. Schobel B. D. Cury J. A. Rosalen P. L. Park Y. K. (2003). Inhibition of *Streptococcus mutans* biofilm accumulation and polysaccharide production by apigenin and tt-farnesol. *J. Antimicrob. Chemother.* 52 782–789. 10.1093/jac/dkg449 14563892

[B26] Koul A. Dendouga N. Vergauwen K. Molenberghs B. Vranckx L. Willebrords R. (2007). Diarylquinolines target subunit c of mycobacterial ATP synthase. *Nat. Chem. Biol.* 3 323–324. 10.1038/nchembio884 17496888

[B27] Kuhnert W. L. Zheng G. Faustoferri R. C. Quivey R. G. (2004). The F-ATPase operon promoter of *Streptococcus mutans* is transcriptionally regulated in response to external pH. *J. Bacteriol.* 186 8524–8528. 10.1128/jb.186.24.8524-8528.2004 15576803 PMC532412

[B28] Lamont R. J. Koo H. Hajishengallis G. (2018). The oral microbiota: dynamic communities and host interactions. *Nat. Rev. Microbiol.* 16 745–759. 10.1038/s41579-018-0089-x 30301974 PMC6278837

[B29] Liu Y. Tang H. Lin Z. Xu P. (2015). Mechanisms of acid tolerance in bacteria and prospects in biotechnology and bioremediation. *Biotechnol. Adv.* 33 1484–1492. 10.1016/j.biotechadv.2015.06.001 26057689

[B30] Marsh P. D. Zaura E. (2017). Dental biofilm: ecological interactions in health and disease. *J. Clin. Periodontol.* 44(Suppl. 18) S12–S22. 10.1111/jcpe.12679 28266111

[B31] Naha P. C. Liu Y. Hwang G. Huang Y. Gubara S. Jonnakuti V. (2019). Dextran-Coated iron oxide nanoparticles as biomimetic catalysts for localized and pH-activated biofilm disruption. *ACS Nano.* 13 4960–4971. 10.1021/acsnano.8b08702 30642159 PMC7059368

[B32] Narang R. Kumar R. Kalra S. Nayak S. K. Khatik G. L. Kumar G. N. (2019). Recent advancements in mechanistic studies and structure activity relationship of FF ATP synthase inhibitor as antimicrobial agent. *Eur. J. Med. Chem.* 182:111644. 10.1016/j.ejmech.2019.111644 31493745

[B33] Pereira-Cenci T. Cenci M. S. Fedorowicz Z. Azevedo M. (2013). Antibacterial agents in composite restorations for the prevention of dental caries. *Cochrane Datab. Syst. Rev.* 12:CD007819. 10.1002/14651858.CD007819.pub3 24343855 PMC8939247

[B34] Peres M. A. Macpherson L. M. D. Weyant R. J. Daly B. Venturelli R. Mathur M. R. (2019). Oral diseases: a global public health challenge. *Lancet (London, Engl.)* 394 249–260. 10.1016/S0140-6736(19)31146-8 31327369

[B35] Pfaller M. A. Diekema D. J. (2012). Progress in antifungal susceptibility testing of *Candida* spp. by use of clinical and laboratory standards institute broth microdilution methods, 2010 to 2012. *J. Clin. Microbiol.* 50 2846–2856. 10.1128/jcm.00937-12 22740712 PMC3421803

[B36] Pitts N. B. Zero D. T. Marsh P. D. Ekstrand K. Weintraub J. A. Ramos-Gomez F. (2017). Dental caries. *Nat. Rev. Dis. Prime.* 3:17030. 10.1038/nrdp.2017.30 28540937

[B37] Pogoryelov D. Krah A. Langer J. D. Yildiz Ö Faraldo-Gómez J. D. Meier T. (2010). Microscopic rotary mechanism of ion translocation in the Fo complex of ATP synthases. *Nat. Chem. Biol.* 6 891–899. 10.1038/nchembio.457 20972431

[B38] Preiss L. Langer J. D. Yildiz Ö Eckhardt-Strelau L. Guillemont J. E. Koul A. (2015). Structure of the mycobacterial ATP synthase Fo rotor ring in complex with the anti-TB drug bedaquiline. *Sci. Adv.* 1:e1500106. 10.1126/sciadv.1500106 26601184 PMC4640650

[B39] Qi M. Chi M. Sun X. Xie X. Weir M. D. Oates T. W. (2019). Novel nanomaterial-based antibacterial photodynamic therapies to combat oral bacterial biofilms and infectious diseases. *Int. J. Nanomed.* 14 6937–6956. 10.2147/ijn.s212807 31695368 PMC6718167

[B40] Sachs G. (1984). Pump blockers and ulcer disease. *New Engl. J. Med.* 310 785–786. 10.1056/nejm198403223101211 6230536

[B41] Saputo S. Faustoferri R. C. Quivey R. G. (2018). A drug repositioning approach reveals that *Streptococcus mutans* is susceptible to a diverse range of established antimicrobials and nonantibiotics. *Antimicrob. Agents Chemother.* 62:1. 10.1128/AAC.01674-17 PMC5740335 29061736

[B42] Selwitz R. H. Ismail A. I. Pitts N. B. (2007). Dental caries. *Lancet (London, Engl.)* 369 51–59. 10.1016/S0140-6736(07)60031-2 17208642

[B43] Spugnini E. Fais S. (2017). Proton pump inhibition and cancer therapeutics: a specific tumor targeting or it is a phenomenon secondary to a systemic buffering? *Semin. Cancer Biol.* 43 111–118. 10.1016/j.semcancer.2017.01.003 28088584

[B44] Takahashi N. Nyvad B. (2011). The role of bacteria in the caries process: ecological perspectives. *J. Dent. Res.* 90 294–303. 10.1177/0022034510379602 20924061

[B45] Teng F. Yang F. Huang S. Bo C. Xu Z. Z. Amir A. (2015). Prediction of early childhood caries via spatial-temporal variations of oral microbiota. *Cell Host Microbe* 18 296–306. 10.1016/j.chom.2015.08.005 26355216

[B46] Xiao J. Klein M. I. Falsetta M. L. Lu B. Delahunty C. M. Yates J. R. (2012). The exopolysaccharide matrix modulates the interaction between 3D architecture and virulence of a mixed-species oral biofilm. *PLoS Pathogens* 8:e1002623. 10.1371/journal.ppat.1002623 22496649 PMC3320608

[B47] Yin I. X. Zhao I. S. Mei M. L. Lo E. C. M. Tang J. Li Q. (2020). Synthesis and characterization of fluoridated silver nanoparticles and their potential as a non-staining anti-caries agent. *Int. J. Nanomed.* 15 3207–3215. 10.2147/ijn.s243202 32440119 PMC7212993

[B48] Yu S. Yun E. J. Kim D. H. Park S. Y. Kim K. H. (2019). Anticariogenic activity of agarobiose and agarooligosaccharides derived from red macroalgae. *J. Agric. Food Chem.* 67 7297–7303. 10.1021/acs.jafc.9b01245 31244198

[B49] Zhang K. Wang S. Zhou X. Xu H. H. Weir M. D. Ge Y. (2015). Effect of antibacterial dental adhesive on multispecies biofilms formation. *J. Dent. Res.* 94 622–629. 10.1177/0022034515571416 25715378 PMC4485219

