# Supplementary material for: In vitro Antibacterial Activity of an FDA-Approved H+-ATPase Inhibitor, Bedaquiline, Against Streptococcus mutans in Acidic Milieus
Source: Front Microbiol. 2021 Feb 25;12:647611. doi: 10.3389/fmicb.2021.647611 (PMC7947916; doi:10.3389/fmicb.2021.647611)
Supplement: Supplementary file 1 [file Data_Sheet_1.docx]

**In vitro antibacterial activity of an FDA-approved H^+^-ATPase inhibitor, bedaquiline, against *Streptococcus mutans* in acidic milieus**

**Meng Zhang ^1,2^, Wenqian Yu ^1^, Shujing Zhou ^3^, Bing Zhang ^1^, Edward Chin Man Lo ^2^, Xin Xu ^1*^, Dongjiao Zhang^1*^.**

^1^ School and Hospital of Stomatology, Cheeloo College of Medicine, Shandong University & Shandong Provincial Key Laboratory of Oral Tissue Regeneration & Shandong Engineering Laboratory for Dental Materials and Oral Tissue Regeneration, Jinan, China; ^2^ Faculty of Dentistry, University of Hong Kong, 34 Hospital Road, Sai Ying Pun, Hong Kong, Hong Kong S.A.R; ^3^ Department of Stomatology, Maternal and Child Health Hospital of Liaocheng city, Liaocheng, China.

*Correspondence:

Xin Xu

[xuxin@sdu.edu.cn](mailto:xuxin@sdu.edu.cn)

Dongjiao Zhang

[djzhang1109@163.com](mailto:djzhang1109@163.com)


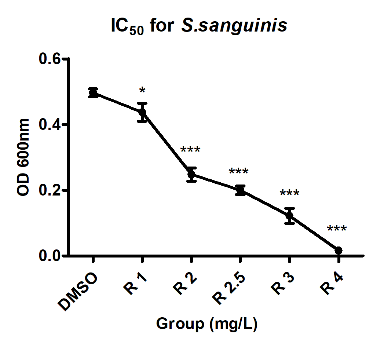


Figure S1: The IC_50_ assay of bedaquiline against planktonic *S.sanguinis* in pH 5 BHI. ^*^, ^**^, *^***^* indicate statistically significant differences at *p* < 0.05, *p* < 0.01, *p* < 0.001, respectively. Error bars are standard deviations.


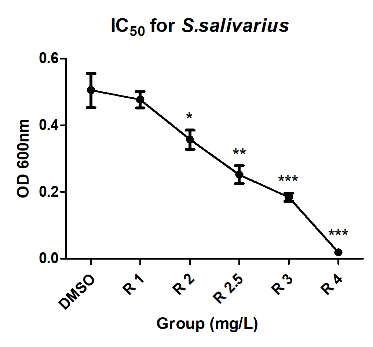


Figure S2: The IC_50_ assay of bedaquiline against planktonic *S.salivarius* in pH 5 BHI. ^*^, ^**^, *^***^* indicate statistically significant differences at *p* < 0.05, *p* < 0.01, *p* < 0.001, respectively. Error bars are standard deviations.


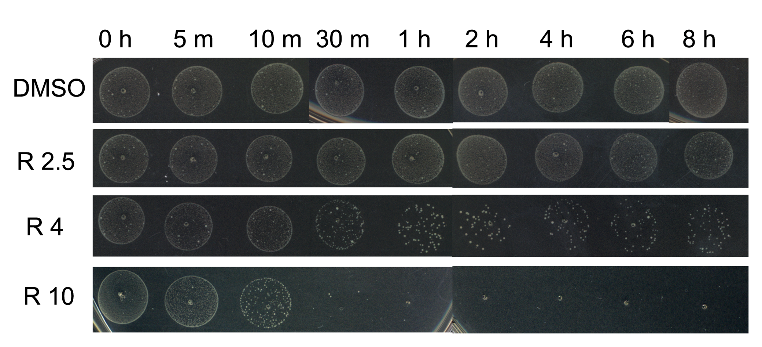


Figure S3: The drop assay of 2.5 mg/L-10 mg/L of bedaquiline and DMSO against planktonic *S.mutans* in pH 5 BHI.


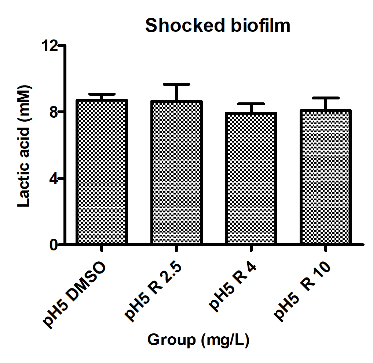


Figure S4: Effect of 2.5 and 10 mg/L of bedaquiline and DMSO on lactic acid production within shocked mature *S.mutans* biofilm.


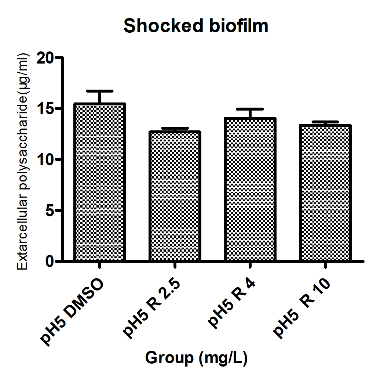


Figure S5: Effect of 2.5 and 10 mg/L of bedaquiline and DMSO on water insoluble extracellular polysaccharide production within shocked mature *S.mutans* biofilm.
